# Antioxidant responses of wheat plants under stress

**DOI:** 10.1590/1678-4685-GMB-2015-0109

**Published:** 2016

**Authors:** Andréia Caverzan, Alice Casassola, Sandra Patussi Brammer

**Affiliations:** 1 Programa Nacional de Pós-Doutorado (PNPD/CNPq), Empresa Brasileira de Pesquisa Agropecuária, Embrapa Trigo, Passo Fundo, RS, Brazil; 2 Programa de Pós-Graduação em Agronomia, Faculdade de Agronomia e Medicina Veterinária (FAMV), Universidade de Passo Fundo, Passo Fundo, RS, Brazil; 3 Empresa Brasileira de Pesquisa Agropecuária, Embrapa Trigo, Passo Fundo, RS, Brazil

**Keywords:** abiotic stress, antioxidant enzymes, hydrogen peroxide signaling, wheat, Triticum aestivum

## Abstract

Currently, food security depends on the increased production of cereals such as wheat (*Triticum aestivum* L.), which is an important source of calories and protein for humans. However, cells of the crop have suffered from the accumulation of reactive oxygen species (ROS), which can cause severe oxidative damage to the plants, due to environmental stresses. ROS are toxic molecules found in various subcellular compartments. The equilibrium between the production and detoxification of ROS is sustained by enzymatic and nonenzymatic antioxidants. In the present review, we offer a brief summary of antioxidant defense and hydrogen peroxide (H_2_O_2_) signaling in wheat plants. Wheat plants increase antioxidant defense mechanisms under abiotic stresses, such as drought, cold, heat, salinity and UV-B radiation, to alleviate oxidative damage. Moreover, H_2_O_2_ signaling is an important factor contributing to stress tolerance in cereals.

## Introduction

In addition to environmental adversities, the world's agriculture faces serious challenges to meet demand, including increased consumption, allocation of land for other uses and the use of chemical products with implications for health safety (Curtis and Halford, 2014). Currently, food security depends on the increased production of mainly three cereals: wheat (*Triticum aestivum* L.), rice (*Oryza sativa* L.) and maize (*Zea mays* L.).

Wheat is one of the major cereals in the world and is one of the main sources of calories and protein. Approximately 85% and 82% of the global population depends on wheat for basic calories and protein, respectively (Chaves *et al.*, 2013). Moreover, this cereal is used in the production of a variety of wheat products, such as leavened bread, flat and steamed breads, cakes, pasta, biscuits, noodles, couscous and beer (Curtis and Halford, 2014). Beyond its use for human consumption, wheat is also used for the development of non-food products such as fuel. Because of its high level of adaptation, wheat is cultivated in tropical and subtropical regions and under both rain-fed and irrigated cultivation. However, crop production is severely affected by adverse environmental stresses (Rahaie *et al.*, 2013).

The main stresses include salt, drought, water excess, UV-B radiation, cold, heat, pathogens, insects, chemicals, ozone, and oil nutrient deprivation (Mahajan and Tuteja, 2005; Cançado, 2011). Under stress plant development and reproduction may be affect at different severity levels, furthermore, the stress is maximized when it occurs in combination.

Environmental stress induces the accumulation of reactive oxygen species (ROS) in the cells, which can cause severe oxidative damage to the plants, thus inhibiting growth and grain yield. The equilibrium between the production and scavenging of ROS is commonly known as redox homeostasis. However, when ROS production overwhelms the cellular scavenging capacity, thus unbalancing the cellular redox homeostasis, the result is a rapid and transient excess of ROS, known as oxidative stress (Mullineaux and Baker, 2010; Sharma *et al.*, 2012). Plants have antioxidant mechanisms for scavenging the ROS excess and prevent damages to cells.

Therefore, this review will address oxidative stress. An overview of the principal antioxidant enzymes involved in wheat plants in ROS detoxification under abiotic stresses, such as drought, cold, heat, salinity and UV-B radiation, will be presented. Furthermore, signaling by ROS in wheat improving stress tolerance will also be covered.

## Antioxidant responses

To avoid potential damage caused by ROS to cellular components, as well as to maintain growth, metabolism, development, and overall productivity, the balance between production and elimination of ROS at the intracellular level must be tightly regulated and/or efficiently metabolized. This equilibrium between the production and detoxification of ROS is sustained by enzymatic and nonenzymatic antioxidants (Mittler, 2002; Mittler *et al.*, 2004).

The enzymatic components comprise several antioxidant enzymes, such as superoxide dismutase (SOD), catalase (CAT), glutathione peroxidase (GPX), guaiacol peroxidase (POX) peroxiredoxins (Prxs), and enzymes of the ascorbate-glutathione (AsAGSH) cycle, such as ascorbate peroxidase (APX), monodehydroascorbate reductase (MDHAR), dehydroascorbate reductase (DHAR), and glutathione reductase (GR) (Asada, 1999; Mittler, 2002, 2004). Nonenzymatic components include the major cellular redox buffers ascorbate (AsA) and glutathione (GSH) as well as tocopherol, carotenoids and phenolic compounds (Mittler *et al.*, 2004; Gratão *et al.*, 2005; Scandalios, 2005).

In wheat, several studies have reported changes in the activity of many enzymes of the antioxidant defense system in plants to control oxidative stress induced by environmental stresses. Alterations in the activity of SOD, APX, CAT, GR and POX and in the ROS concentration were reported in wheat plants in field and laboratory conditions (Srivalli and Khanna-Chopra, 2001;Kocsy *et al.*, 2002;Špundová *et al.*, 2005;Varga *et al.*, 2012;Mishra *et al.*, 2013;Rao *et al.*, 2013; Huseynova *et al.*, 2014, Kong *et al.*, 2014; Talaat and Shawky, 2014, Wang *et al.*, 2008). These studies demonstrated that, in wheat, the mechanisms of ROS detoxification are positively activated. Several studies showed that, in an attempt to defend itself against oxidative damage, wheat plants under different abiotic stresses alter the activity of antioxidant enzymes such as SOD, CAT, APX, POX and GR (Table 1).

**Table 1:** Summary of the antioxidant enzyme changes in different wheat genotypes and different tissues type under different tested abiotic-stress conditions.

Abiotic stress	SOD	CAT	APX	POX	GR	References
Drought	↓	-	-	↓		Alexieva *et al*. (2001)
		↑				Luna *et al*. (2005)
	-	↑	↑	↑	↑	Devi *et al*. (2012)
	↑	↑	↑		↑	Wang *et al*. (2008)
Salinity		↑	↑	↑		Barakat (2011)
		↑	↓	↓		Heidari (2009)
	↑	↑			↓	Esfandiari *et al*. (2007)
	↑	↑			↑	Sairam *et al*. (2002)
Cold	↑	↑	↑	↑		Janmohammadi *et al*. (2012)
	↑	-	↑	↑	↑	Turk *et al*. (2014)
Heat	↑	↑	↑		↑	Badawi *et al*. (2007)
	↑			↑		Ibrahim *et al*. (2013)
	↑	↑		↑		Gupta *et al*. (2013)
	↓↑	↓		↓	↓↑	Wang *et al*. (2014)
UV-B	↑			↑		Ibrahim *et al*. (2013)
	↑	↑		-		Alexieva *et al*. (2001)
	↓	↓		↓	↑	Barabás *et al*. (1998)

Superoxide dismutase (SOD), catalase (CAT), ascorbate peroxidase (APX), guaiacol peroxidase (POX) and glutathione reductase (GR). (↑) increase, (↓) decline and (-) unchanged.

Excess ROS is harmful to the plant; thereby, to restore the cellular redox balance, both enzymatic and nonenzymatic systems are activated to detoxify the toxic levels of ROS. In response to the major abiotic stresses faced by wheat plants, most antioxidant enzymes increased their activity (Table 1). Furthermore, many reports demonstrated that the effect of abiotic stress in wheat is genotype-specific, where some genotypes showed different responses in the same stress condition. Tolerant genotypes generally maintained a higher antioxidant capacity resulting in lower oxidative damage. This property likely depends on the genetic potential of the genotype. Wheat responses also depend on the tissue type, length and intensity of the stress as well as on developmental stage proving the complexity of the mechanisms of production and detoxification of ROS and the effect of ROS (oxidative stress) on antioxidant systems. Many studies have reported an increase in the concentration of hydrogen peroxide (H_2_O_2_) after exposure to a stress, and its production is dependent on the intensity and duration of the stress. Furthermore, the H_2_O_2_ level differs between various cellular compartments and is related to the type of stress (Slesak*et al.*, 2007).

The observed increase in enzymatic activities and decrease in oxidative damage are closely related. The expression of many antioxidant enzymes is positively correlated with higher tolerance levels against abiotic stresses. The activation of some enzymes leads to plant protection against oxidative damage. In rice plants, an important cereal model, increased expression levels of antioxidant enzymes and genes have been related to the response to stress factors (Rosa *et al.*, 2010; Bonifacio *et al.*, 2011; Ribeiro *et al.*; 2012; Caverzan *et al.*, 2014; Passaia *et al.*, 2013, 2014). In another model plant, *Arabidopsis*, the involvement of at least 152 genes was observed in the regulation of the ROS level under stress (Mittler, 2004). Thereby, a complex enzymatic system has evolved in plants to scavenge excess ROS and to protect the plants from oxidative stress.

For example, in wheat, an increase in the *SOD* transcript was observed in response to differential heat shock treatment (Kumar *et al.*, 2013), which indicates an enhanced tolerance to environmental stresses. Superoxide dismutases constitute a frontline in the defense against ROS, they catalyze the dismutation of ${\text{O}}_{2}^{\bar{•}}$ (superoxide radical) to H_2_O_2_. These enzymes are classified according to their subcellular location and metal cofactor (Cu/Zn, Mn, Fe and Ni), and are present in plants, bacteria, yeast and animals. In plants, the SOD genes are regulated by development, tissue-specific and environmental signals (Scandalios, 1997; 2005; Menezes-Benavante *et al.*, 2004).

The wheat *Cat* gene expressed in transgenic rice improves tolerance against low-temperature stress when compared to non-transgenic plants (Matsumura *et al.*, 2002). Catalases remove the H_2_O_2_, reducing H_2_O_2_to 2H_2_O. These proteins are abundantly, but not exclusively, localized to peroxisomes. The CATs genes respond differentially to various stresses conditions (Scandalios, 2002; 2005).

In wheat, a mutant line with reduced thylakoid *APX* activity leads to impaired photosynthesis (Danna *et al.*, 2003). Rice mutants double silenced for cytosolic*APX*s exhibit high guaiacol peroxidase activity, which can contribute to the cytosolic H_2_O_2_ scavenging that occurs in the vacuoles or apoplast (Bonifacio *et al.*, 2011). Ascorbate peroxidases catalyze the conversion of H_2_O_2_ into H_2_O and use ascorbate as a specific electron donor. APX proteins are distributed in chloroplasts, mitochondria, peroxisomes and the cytosol. The *APX* genes show differential modulation by several abiotic stresses in plants (Rosa *et al.*, 2010; Caverzan *et al.*, 2012; Caverzan *et al.*, 2014). The balance between SODs, CATS and APXs is important for determining the intracellular level of ROS, besides changes in the balance of these appear to induce compensatory mechanisms (Apel and Hirt, 2004; Scandalios, 2002; 2005).

Recently, it was demonstrated that knockdown of the wheat*monodehydroascorbate reductase* gene resulted in improved wheat resistance to stripe rust by inhibiting sporulation in the compatible interaction. Moreover, silenced wheat plants increased the proportion of necrotic area at the infection sites and suppressed *Puccinia striiformis* f. sp.*tritici* hypha elongation (Feng*et al.*, 2014). Monodehydroascorbate reductase catalyze the regeneration of AsA from the monodehydroascorbate radical using NAD(P)H as an electron donor. Thereby, MDHAR in the plant antioxidant system maintains the AsA pool (Hossain and Asada, 1985). Isoforms of MDHAR are present in chloroplasts, cytosol, peroxisomes and mitochondria (Jiménez *et al.*, 1997; Leterrier *et al.*, 2005).

The expression of wheat *GPX* genes was altered when wheat plants were submitted to salt, H_2_O_2_ and abscisic acid treatment. Moreover, other findings suggest that *GPX* genes not only act as scavengers of H_2_O_2_ to control abiotic stress responses but also play important roles in salt and ABA-signaling cascades (Zhai *et al.*, 2013). In addition, glutathione peroxidases studies have demonstrated that *GPX* genes are essential for redox homeostasis in rice (Passaia *et al.*, 2013, 2014). Glutathione peroxidases catalyze the reduction of H_2_O_2_ or organic hydroperoxides to water. The GPXs proteins are present in many life species (Margis *et al.*, 2008). In plants, the GPX proteins are distributed in mitochondria, chloroplasts and the cytosol.

## Signaling by H_2_O_2_

ROS are well recognized for playing a dual role, both as deleterious as well as beneficial, depending on their concentration in plants. The role of ROS as signaling molecules involved in processes such as growth, cell cycle, development, senescence, programmed cell death, stomatal conductance, hormonal signaling, and regulation of gene expression has been widely explored (Kovtun*et al.*, 2000; Neill*et al.*, 2002; Slesak*et al.*, 2007; Inze*et al.*, 2012). The intensity and duration of ROS signaling also depends on the pool that results due to the production of ROS by oxidants and their removal by antioxidants (Sharma*et al.*, 2012). Among the various ROS, H_2_O_2_ is one of the most abundant in aerobic biological systems in higher plants, being highly reactive and toxic. Hydrogen peroxide is considered a signaling molecule in plants that mediates responses to various biotic and abiotic stresses. The biological effect of H_2_O_2_ is related to several factors, such as the site of production, the developmental stage of the plant, and previous exposures to different kinds of stress, however the strongest effect on plants is the relationship with its concentration (Petrov and Breusegem, 2012).

Hydrogen peroxide can diffuse across cell membranes and be transported to other compartments, where it can act as a signaling molecule or be eliminated (Neill *et al.*, 2002). Thus, due the property that in low concentrations the H_2_O_2_ acts as stress signal, many studies have demonstrated that its application can induce stress tolerance in plants. Low H_2_O_2_ treatments improve seed germination, seedling growth and resistance to abiotic stresses.

In wheat, it was observed that seed pretreatment with H_2_O_2_enhances drought tolerance of seedlings (He*et al.*, 2009). Moreover, H_2_O_2_pretreatment improved wheat aluminum acclimation during subsequent aluminum exposure, thereby reducing ROS accumulation (Xu*et al.*, 2011). The exogenous H_2_O_2_ treatment also protected wheat seedlings from damage by salt stress (Li *et al.*, 2011), and the pretreatment of seeds enhanced salt tolerance of wheat seedlings, decreasing the oxidative damage (Wahid*et al.*, 2007). Maize plants originated from H_2_O_2_ pretreated seeds showed increased tolerance to salt stress (Gondim *et al.*, 2010). In rice plants, H_2_O_2_ not only acts as a toxic molecule but also as a signaling molecule associated with salinity, cadmium and abscisic acid stresses (Kao, 2014).

Considerable evidence suggests that H_2_O_2_ and other ROS may act as important signal molecules mediating response to stress tolerance in plants (Neill *et al.*, 2002). Although, recent studies have demonstrated that in wheat and others plant species the H_2_O_2_ treatment enhances tolerance to different stresses, these responses are poorly explored in later stages of growth and even adult plants. Physiological responses of the plant can vary according with the stage of development. Besides, in wheat it was demonstrated that H_2_O_2_plays two important roles, one as a signal molecule and other as a harmful chemical, when wheat seedlings were grown under H_2_O_2_ stress (Ge *et al.*, 2013). Thus, the H_2_O_2_ concentration, low or high, will determine whether the effect will be deleterious or beneficial in plants.

Petrov and Breusegem (2012) showed the major signaling components in the H_2_O_2_-transduction network, their interactions and different outcomes in the plant cell. These include transcription factors, miRNAs, MAP-kinases and the interaction of the some effects. In addition, the H_2_O_2_ concentration, site of H_2_O_2_synthesis, interaction with other active signaling pathways, previous exposure to stress, etc, are also important.

Thus, the mechanism by which a ROS treatment may protect against different stresses needs to be further investigated because other pathways (biochemical, molecular and genetic) can be involved and contribute to tolerance. Importantly, each plant species responds differently to stress condition and under field conditions, and oftentimes the plants suffer combined stresses. However, ROS signaling mechanisms is potentially significant to any program aimed at improving crop tolerance to environmental stresses.

## Final considerations

In the present review we list evidence that wheat plants activate antioxidant defense mechanisms under abiotic stresses, which helps in maintaining the structural integrity of the cell components and presumably alleviates oxidative damage. Moreover, H_2_O_2_ signaling can contribute to wheat plant tolerance to environmental stresses. However, this route must be further explored, as many enzymes and isoforms can be involved, and ROS is only one of the potential parameters of plant biological tolerance against environmental variations.
